# Persistent CD8^+^ T cell-driven immune dysregulation despite normalized CD4^+^ T cell recovery in ART-treated people living with HIV

**DOI:** 10.3389/fimmu.2026.1735779

**Published:** 2026-02-09

**Authors:** Yiyao Hu, Lingyun Ge, Yun He, Xiaorui Li, Yinsong Luo, Hui Wu, Jiayi He, Chao Zhang, Jiaye Liu

**Affiliations:** 1 School of Public Health, Shenzhen University Medical School, Shenzhen, China; 2 Biobank, Shenzhen Children’ s Hospital, Shenzhen, China; 3 Department of Infectious Diseases, National Clinical Research Center for Infectious Diseases, Shenzhen Third People’s Hospital, Shenzhen, China; 4 Senior Department of Infectious Diseases, The Fifth Medical Centre of Chinese PLA General Hospital, National Clinical Research Center for Infectious Diseases, Beijing, China

**Keywords:** antiretroviral therapy, CD4/CD8 ratio, CD8+ T cells, HIV, immune dysregulation

## Abstract

**Background:**

Despite successful antiretroviral therapy (ART) that restores CD4^+^ T cell counts and reduces HIV viral loads to undetectable levels, a substantial proportion of people living with HIV (PLWH) exhibit persistent CD4/CD8 ratio inversion. This abnormal ratio is primarily driven by sustained CD8^+^ T cell expansion and reflects a state of chronic immune dysregulation and incomplete immune recovery. However, cellular and molecular mechanisms underlying this discordant immune state remain poorly understood.

**Methods:**

We analyzed longitudinal data of 5,416 ART-treated PLWH from Shenzhen Third People’s Hospital, identifying distinct CD8^+^ T cell trajectory groups using group-based trajectory modeling. We compared those with chronic stable activation (CSA group) versus those with immune modulation recovery (IMR group) using CyTOF-based immunophenotyping, bulk RNA sequencing, and plasma biomarker profiling.

**Results:**

Both IMR and CSA groups achieved CD4^+^ T cell recovery, but CSA group exhibited persistently elevated CD8^+^ T cells and inverted CD4/CD8 ratios. The CSA group displayed a marked expansion of senescent and activated CD8^+^ T cell subsets and diminished regulatory T cells, characterized by decreased expression of CD196, CD95, and CD27. Bulk RNA sequencing revealed upregulation of interferon-stimulated genes, chemokine signaling pathways and pro-inflammatory transcriptional programs. Consistently, systemic levels of key inflammatory mediators, including IP-10, MCP-1, and soluble CD163, were significantly elevated in the CSA group.

**Conclusions:**

Persistent CD8^+^ T cell activation reflects a distinct immunological state marked by CD4/CD8 ratio inversion, cell senescence, exhaustion, and systemic inflammation. This immune profile may help identify individuals who warrant closer immunological monitoring for non-AIDS complications and may inform future studies aimed at modulating CD8^+^ T cell-driven immune dysregulation to improve long-term immune restoration.

## 1 Introduction

Human immunodeficiency virus (HIV) infection leads to acquired immune deficiency syndrome (AIDS), a systemic disease that remains a major public health challenge. Although antiretroviral therapy (ART) effectively suppresses viral replication and restores CD4^+^ T cell counts in people living with HIV (PLWH) (1, 2), many individuals fail to achieve complete immune restoration, as indicated by persistently elevated CD8^+^ T cell counts and inverted CD4/CD8 ratios despite virological suppression (3, 4).

Mounting evidence suggests that a low CD4/CD8 ratio and sustained CD8^+^ T cell expansion are associated with increased risk of non-AIDS-related comorbidities, including cardiovascular disease, liver disease, and cancer (3, 5–7). These immune abnormalities are not captured by CD4^+^ T cell counts alone and likely reflect residual immune alterations that persist beyond viral control (8), which may be driven by multiple factors, including low-level expression from latent viral reservoirs, gut microbial translocation, and reactivation of co-infections such as cytomegalovirus (CMV)—all of which can contribute to chronic CD8^+^ T cell stimulation. Sustained activation leads to CD8^+^ T cell exhaustion, heightened expression of inhibitory receptors (e.g., programmed cell death protein-1 [PD-1], T cell immunoglobulin and mucin domain-containing protein-3 [TIM-3]) (9, 10), inflammatory cytokines (e.g., Interleukin-6 [IL-6], C-reactive protein [CRP]) (11, 12), and monocyte/macrophage markers (e.g., CD14, CD163) (13, 14). These immune perturbations underlie a chronic inflammatory state linked to non-AIDS complications, even in the setting of viral suppression (15–19).

While the prognostic implications of CD8^+^ T cell elevation and CD4/CD8 ratio inversion are increasingly recognized, they are pivotal in the immune surveillance of PLWH (20). The underlying cellular and molecular mechanisms remain poorly defined. Prior studies have primarily focused on clinical endpoints, with limited characterization of the phenotypic and functional features of CD8^+^ T cells or the associated inflammatory milieu in PLWH with discordant immune recovery. Most studies relied on single-timepoint measurements and lacked high-dimensional immune profiling. Few have leveraged longitudinal immune data or group-based trajectory modeling (GBTM) to define patterns of immune recovery in ART-treated individuals. Despite considerable research efforts, the phenotypic and transcriptional features of CD8^+^ T cell-driven dysregulation under long-term ART remain largely undefined. Determining the role of CD4/CD8 ratio or CD8^+^ count as new prognostic markers would help identify those patients at high risk for morbidity and mortality.

In this study, we leveraged a large ART-treated cohort and employed GBTM to stratify individuals based on CD8^+^ T cell trajectories. Using mass cytometry (CyTOF), bulk RNA sequencing, and plasma biomarker profiling, we aimed to delineate the immunological landscape of patients with persistently low CD4/CD8 ratios despite CD4^+^ T cell recovery, providing mechanistic insights into CD8^+^ T cell-driven immune dysregulation.

## 2 Methods

### 2.1 Study design and participants

**Figure 1:**
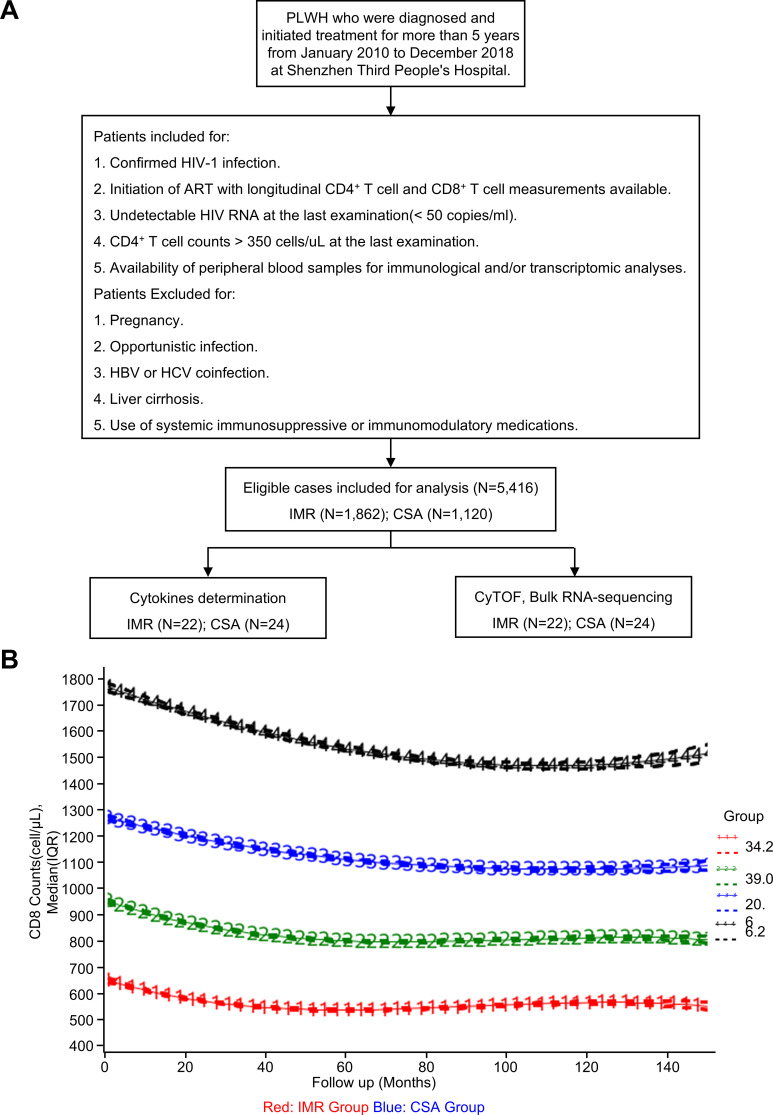
Flow chart of participants enrolled in this study. **(A)** Flow chart of participants enrolled in this study. A total of 5,416 PLWH were included in this study. A total of 46 participants (IMR, n = 22; CSA, n = 24) were selected for plasma cytokines determination, and a nested subset of 10 PLWH (IMR, n = 5; CSA, n = 5) was further selected for both CyTOF and bulk RNA-sequencing. **(B)** The approach implemented by GBTM was used to identify subgroups within each group that shared a similar underlying trajectory of CD8^+^ T cell counts (Total = 5,416; IMR, n = 1,862; CSA, n = 1,120). The red curve indicates IMR and the blue curve indicates CSA. IMR, Immune Modulation Recovery; CSA, Chronic Stable Activation.

We conducted a retrospective cohort study of PLWH who had received ART for at least five years between January 2010 and December 2018 at Shenzhen Third People’s Hospital. Data collection was censored in May 2024. A flowchart detailing patient selection and exclusion criteria is shown in **Figure 1A**. These criteria were applied to minimize confounding factors that could influence host immune response or affect the size of the HIV reservoir. All participants provided written informed consent prior to any procedures. Eligibility was confirmed, and detailed medical history and concomitant medications were documented. After applying the specified screening parameters, 5,416 PLWH met the study inclusion criteria.

Demographic and clinical data were collected, including sex, age at ART initiation, route of HIV infection, HIV RNA levels, baseline CD4^+^ and CD8^+^ T cell counts, baseline CD4/CD8 ratio, and ART regimen. Concurrently, the latest plasma samples were obtained and stored at -80°C for subsequent analyses.

### 2.2 Group-based trajectory modelling

We applied GBTM using the Proc Traj procedure in SAS (SAS Institute Inc., Cary, North Carolina, USA) to identify subgroups of individuals with similar longitudinal patterns of CD8^+^ T cell counts (**Figure 1b**, **Supplementary Figure S1**). Prior to model fitting, we performed likelihood ratio tests to determine the best-fit polynomial function (linear, quadratic, or cubic) for each trajectory. Model selection was guided by Bayesian information criterion (BIC), average posterior probabilities of group membership (>0.7), and sufficient representation within each subgroup (>5% of the cohort) (**Supplementary Table S1**).

### 2.3 Plasma HIV RNA, CD4^+^ T cell, and CD8^+^ T cell counts determination

CD4^+^ T cell and CD8^+^ T cell counts were quantified by flow cytometry. Plasma HIV RNA levels were measured using the COBAS AmpliPrep/TaqMan real-time RT-PCR assay. HIV RNA was amplified targeting the gag region using the following primers and probe: forward primer 5’-GACATAAGACAGGGACCAAAGG-3’; reverse primer 5’-CTGGGTTTGCATTTTGGACC-3’; and probe primer 5’AACTCTAAGAGCCGAGCAAGCTTCAC-3’.

### 2.4 Cytokines determination

Fatty acid-binding protein 2 (FABP2) was detected using a double-antibody sandwich enzyme-linked immunosorbent assay (ELISA). Interferon-α (IFN-α), interferon-γ (IFN-γ), interleukin-15 (IL-15), interleukin-16 (IL-16), interferon-γ-induced protein 10 (IP-10), monocyte chemoattractant protein-1 (MCP-1), CD14, CD163, and CRP were quantified using a high-throughput multiplex bead-based immunoassay. For visualization, cytokine concentrations were transformed using log (x + 1) and then standardized by z-score for each cytokine across all participants. For group comparisons, the original concentrations were used.

### 2.5 CyTOF sample preparation and data acquisition

The 42-metal-conjugated antibodies used in this study are listed in **Supplementary Table S2**. Cryopreserved samples were rapidly thawed and assessed for cell count and viability using trypan blue exclusion. PBMCs were then stained with cisplatin to identify dead cells, washed, and resuspended in cell staining buffer. Fc receptors were blocked using human TruStain FcX, followed by incubation with a cocktail of metal-conjugated surface antibodies (**Supplementary Table S2**). After washing, cells were fixed with paraformaldehyde, and subsequently incubated overnight at 4 °C with a DNA intercalator.

Cells were then resuspended in freshly prepared Cell Acquisition Solution containing 10% EQ Four Element Calibration Beads, and filtered through a cell strainer cap tube. Data acquisition was performed on a CyTOF instrument at PLT TECH (Hangzhou, China).

### 2.6 CyTOF data processing

PBMC samples were manually gated in Cytobank to exclude normalization beads, cell debris, dead cells, and doublets to identify CD45^+^ cisplatin-negative cells for further downstream analysis. We loaded FCS files into R software (version 4.4.0). Signal intensities and each channel marker expression were arcsinh transformed with a cofactor of 5 (x_transf = asinh (x/5)). To visualize the high-dimensional data, t-distributed stochastic neighbor embedding (t-SNE) and the PhenoGraph clustering algorithm were performed on all samples. For t-SNE, 10,000 cell events per sample were pooled, with a perplexity of 30 and a theta of 0.5, using the Barnes-Hut implementation in the R Rtsne package. Visualization was performed using the ggplot2 package.

Marker expression values were normalized between 0 and 1 for display on t-SNE plots using the following formula: Y = (value-minimum)/(maximum-minimum), where the minimum and maximum were defined as the 3rd and 97th percentiles, respectively. Values below 0 were set to 0, and those above 1 were set to 1. Clustering was performed using PhenoGraph (k = 30) on all pooled samples to define cell populations. Heatmaps were generated in R using the ComplexHeatmap package, with marker expression normalized as described above.

### 2.7 RNA isolation

Total RNA was extracted from PBMCs obtained from the 10 PLWH. 200 µL thawed PBMCs was transferred into a microcentrifuge tube, followed by the addition of Lysis Reagent. The mixture was vortexed and incubated at room temperature. Subsequently, chloroform was added to a microcentrifuge tube, vigorously vortexed, and incubated at room temperature. The sample was centrifuged, and the upper aqueous phase was transferred to a new tube. An equal volume of absolute ethanol was added and mixed thoroughly. The resulting solution was applied in aliquots to a RNeasy MinElute spin column, followed by centrifugation. The column was washed with RWT buffer, then twice with RPE buffer, each followed by centrifugation. A final centrifugation was performed to dry the column. The spin column was then placed in a new microcentrifuge tube, and RNase-free water was added directly to the membrane. After incubation at room temperature, RNA was eluted by centrifugation. RNA concentration and integrity were assessed using an Agilent 2100 Bioanalyzer.

### 2.8 mRNA library preparation and bulk RNA-sequencing

Bulk RNA-sequencing was performed on PBMCs obtained from 10 PLWH. First-strand cDNA synthesis was performed using random hexamer-primed reverse transcription, followed by second-strand synthesis to generate double-stranded cDNA. The cDNA underwent end-repair, and a single adenine (A) nucleotide was added to the 3’ ends. Adaptors were ligated to the A-tailed cDNA fragments, and the ligated products were amplified by PCR and subjected to quality control. Subsequently, the amplified double-stranded library was denatured to obtain single-stranded DNA, which was circularized to form single-stranded circular DNA molecules. Uncyclized linear DNA was enzymatically digested. The circularized DNA molecules were then amplified using phi29 DNA polymerase through rolling circle amplification to generate DNA nanoballs, each containing more than 300 copies of the original template. The DNBs were loaded onto a patterned nanoarray, and paired-end 100 bp reads were generated using the G400 sequencing platform by BGI (Shenzhen, China).

### 2.9 Bulk RNA-sequencing data analyses

The sequencing data were filtered using SOAPnuke to obtain high-quality clean reads by (1) removing reads containing sequencing adapters; (2) removing reads with more than 20% low-quality bases (base quality ≤ 15%); and (3) removing reads with more than 5% unknown bases (‘N’).

Clean reads were aligned to the reference genome using HISAT2. Fusion gene detection and differential splicing gene (DSG) analysis were performed using EricScript (version 0.5.5) and rMATS (version 4.1.2), respectively. Bowtie2 was used to map reads to a comprehensive gene set, including known and novel, coding and noncoding transcripts. Gene expression levels were quantified using RSEM (version 1.3.1). Differentially expressed genes (DEGs) were identified using DESeq2 (version 1.34.0) with a significance threshold of P ≤ 0.05 or false discovery rate (FDR) ≤ 0.001. To gain insight into the change of phenotype, Gene Ontology (GO; http://www.geneontology.org/) and Kyoto Encyclopedia of Genes and Genomes (KEGG; https://www.kegg.jp/) enrichment analyses were performed using the Phyper function based on the hypergeometric test.

### 2.10 Statistics

Data were assessed for normality using the Shapiro–Wilk test. Parametric tests were applied only when all groups met the assumption of normal distribution. Statistical analyses were conducted using SPSS (version 27.0.1). For comparisons between the two groups, two-sided non-parametric tests were used, including the Mann–Whitney U test. Correlations were evaluated using Spearman’s rank correlation coefficient. Graphical illustrations and statistical plots were generated using GraphPad Prism (version 9). De-identified, individual-level raw values underlying the main figures are provided in the **Supplementary Tables**. Where applicable, P values from *post hoc* tests were indicated in figure captions. Significance was indicated by *P < 0.05, **P < 0.01, ***P < 0.001, and ****P < 0.0001.

## 3 Results

### 3.1 Characteristics of the study cohort

Among 5,416 ART-treated PLWH who met the inclusion criteria, demographic, clinical, and immunologic characteristics of the study cohort are summarized in **Table 1**.

**Table 1:** Characteristics of PLWH according to the CD8^+^ T cell and Trajectory−Group.

Characteristics^a^	Overall^b^	IMR	CSA	*p* ^c^
N	5416	1862	1120	
Sex				<0.001
Male	4878 (90.1)	1620 (87.0)	1031 (92.1)	
Female	538 (9.9)	242 (13.0)	89 (7.9)	
Age, years	31.00 [26.00, 38.00]	33.00 [27.00, 41.00]	31.00 [26.00, 37.00]	<0.001
BMI, kg/m2				0.029
<18.5	760 (14.0)	273 (14.7)	147 (13.1)	
18.5-23.9	3723 (68.7)	1282 (68.9)	759 (67.8)	
>=24	933 (17.2)	307 (16.5)	214 (19.1)	
Marital status				<0.001
Never married	3175 (58.6)	931 (50.0)	725 (64.7)	
Married or cohabiting	1767 (32.6)	730 (39.2)	301 (26.9)	
Divorced, separated, or widowed	474 (8.8)	201 (10.8)	94 (8.4)	
HIV transmission route				<0.001
Male-to-male sex contact	3543 (65.4)	1162 (62.4)	759 (67.8)	
Heterosexual contact	1742 (32.2)	661 (35.5)	338 (30.2)	
IDU	37 (0.7)	9 (0.5)	4 (0.4)	
Other	94 (1.7)	30 (1.6)	19 (1.7)	
Smoking	1247 (23.0)	424 (22.8)	261 (23.3)	0.967
Drinking	1475 (27.2)	498 (26.7)	308 (27.5)	0.921
HIVRNA, copies/mL				<0.001
<5000	379 (7.0)	145 (7.8)	58 (5.2)	
5000-99999	1708 (31.5)	649 (34.9)	315 (28.1)	
>=100000	3329 (61.5)	1068 (57.4)	747 (66.7)	
Baseline CD4 count, cells/μL				<0.001
<200	1527 (28.2)	527 (28.3)	340 (30.4)	
200-349	2445 (45.1)	909 (48.8)	465 (41.5)	
350-499	1097 (20.3)	349 (18.7)	230 (20.5)	
>=500	347 (6.4)	77 (4.1)	85 (7.6)	
Baseline CD8 count, cells/μL				<0.001
<500	610 (11.3)	468 (25.1)	25 (2.2)	
500-999	2785 (51.4)	1178 (63.3)	369 (32.9)	
>=1000	2021 (37.3)	216 (11.6)	726 (64.8)	
Baseline CD4/CD8 ratio				<0.001
<0.1	350 (6.5)	35 (1.9)	127 (11.3)	
0.1-0.39	3476 (64.2)	905 (48.6)	863 (77.1)	
0.4-0.79	1434 (26.5)	794 (42.6)	129 (11.5)	
>=0.8	156 (2.9)	128 (6.9)	1 (0.1)	
Last CD4 count, cells/μL				<0.001
350-499	1481 (27.3)	637 (34.2)	244 (21.8)	
>=500	3935 (72.7)	1225 (65.8)	876 (78.2)	
Last CD8 count, cells/μL				<0.001
<500	610 (11.3)	468 (25.1)	25 (2.2)	
500-999	2785 (51.4)	1178 (63.3)	369 (32.9)	
>=1000	2021 (37.3)	216 (11.6)	726 (64.8)	
Last CD4/CD8 ratio				<0.001
0.1-0.39	2021 (37.3)	216 (11.6)	726 (64.8)	
0.4-0.79	2021 (37.3)	216 (11.6)	726 (64.8)	
>=0.8	2986 (55.1)	1515 (81.4)	309 (27.6)	
Glucose, mmol/L	5.01 [4.70, 5.34]	5.05 [4.74, 5.39]	5.00 [4.70, 5.33]	<0.001
WBC, 10^9/L	5.26 [4.36, 6.35]	4.90 [4.10, 5.85]	5.61 [4.68, 6.72]	<0.001
Platelet, 10^9/L	204.00 [171.00, 241.00]	199.00 [169.00, 234.00]	211.00 [174.00, 248.00]	<0.001
Low-density lipoprotein cholesterol, mmol/L	2.47 [2.07, 2.92]	2.48 [2.07, 2.94]	2.46 [2.04, 2.89]	0.396
High-density lipoprotein cholesterol, mmol/L	1.26 [1.08, 1.45]	1.30 [1.12, 1.51]	1.23 [1.04, 1.41]	<0.001
Triglycerides, mmol/L	1.22 [0.87, 1.79]	1.14 [0.83, 1.64]	1.28 [0.90, 1.90]	<0.001
Total cholesterol, mmol/L	4.14 [3.64, 4.70]	4.20 [3.69, 4.79]	4.10 [3.54, 4.65]	0.002
Creatinine, μmol/L	72.00 [64.00, 79.00]	72.00 [64.00, 79.00]	72.00 [65.00, 80.00]	0.365
Alanine aminotransferase, U/L				<0.001
>=1ULZ	554 (10.2)	172 (9.2)	125 (11.2)	
>=2ULZ	168 (3.1)	39 (2.1)	40 (3.6)	
Normal	4694 (86.7)	1651 (88.7)	955 (85.3)	
Aspartate aminotransferase, U/L				<0.001
>=1ULN	325 (6.0)	92 (4.9)	93 (8.3)	
>=2ULN	91 (1.7)	20 (1.1)	17 (1.5)	
Normal	5000 (92.3)	1750 (94.0)	1010 (90.2)	
Diabetes	107 (2.0)	41 (2.2)	22 (2.0)	0.8
Hypertension	52 (1.0)	20 (1.1)	5 (0.4)	0.249
Opportunistic Infections	318 (5.9)	64 (3.4)	93 (8.3)	<0.001
Time interval				<0.001
<1	2147 (39.6)	793 (42.6)	405 (36.2)	
1-5	1632 (30.1)	607 (32.6)	328 (29.3)	
>=6	1637 (30.2)	462 (24.8)	387 (34.6)	
HBV infection	590 (11.9)	214 (12.4)	100 (10.0)	0.205
HCV infection	82 (1.6)	22 (1.3)	19 (1.9)	0.27
WHO Stage				<0.001
I	1284 (23.7)	381 (20.5)	282 (25.2)	
II	2457 (45.4)	913 (49.0)	457 (40.8)	
III	878 (16.2)	348 (18.7)	187 (16.7)	
IV	797 (14.7)	220 (11.8)	194 (17.3)	
ART treatment regimen				<0.001
3TC+TDF+EFV/NVP	2915 (53.8)	1105 (59.3)	535 (47.8)	
DTG-containing	1200 (22.2)	332 (17.8)	278 (24.8)	
3TC/AZT+EFV/NVP/LPV/r	523 (9.7)	177 (9.5)	116 (10.4)	
3TC+LPV/r+TDF/AZT/D4T	444 (8.2)	132 (7.1)	113 (10.1)	

IMR, immune modulation recovery; CSA, chronic stable activation; BMI, body-mass index; IDU, injection drug use; WBC, white blood cells; Time interval, the time between the diagnosis of HIV and the initiation of ART; HBV, hepatitis B virus; HCV, hepatitis C virus; HIV, human immunodeficiency virus; ART, antiretroviral therapy; 3TC, lamivudine; TDF, tenofovir disoproxil fumarate; EFV, efavirenz; NVP, nevirapine; DTG, Dolutegravir; AZT, zidovudine; LPVr, lopinavir/ritonavir; D4T, stavudine; EVG, elvitegravir; FTC, emtricitabine; TAF, tenofovir alafenamide. ^a^Continuous variables are expressed as median (IQR). ^b^Categorical variables are expressed as frequency (percentage). ^c^Significant differences are indicated by *P* < 0.05. Differences between each group were analyzed including one-way ANOVA for continuous variables and the chi-square test for categorical variables.

To further characterize longitudinal immune dynamics, we applied GBTM to identify the optimal trajectory of CD8^+^ T cell counts in the cohort. According to BIC and AIC value comparisons, a four-group model was selected as optimal, balancing model complexity with data fit (**Figure 1B**, **Supplementary Table S1**). Alternative grouping results are shown in **Supplementary Figure S1**, **Supplementary Table S1**. Based on the four CD8^+^ T cell trajectories identified by GBTM, we chose the two trajectories with larger sample sizes, opposite dynamic patterns, and the greatest divergence in CD8^+^ T cell means for subsequent analysis. These were classified as the chronic stable activation (CSA, blue, n = 1,120) and immune modulation recovery (IMR, red, n = 1,862) groups. The CSA group was characterized by steadily increasing CD4^+^ T cell counts, persistently elevated and relatively stable CD8^+^ T cell counts, and a continuously low CD4/CD8 ratio (<0.8). In contrast, the IMR subgroup showed progressively increasing CD4^+^ T cell counts, declining CD8^+^ T cell counts, and a corresponding rise in the CD4/CD8 ratio (≥0.8) during follow-up (**Figure 1B**).

**Figure 2:**
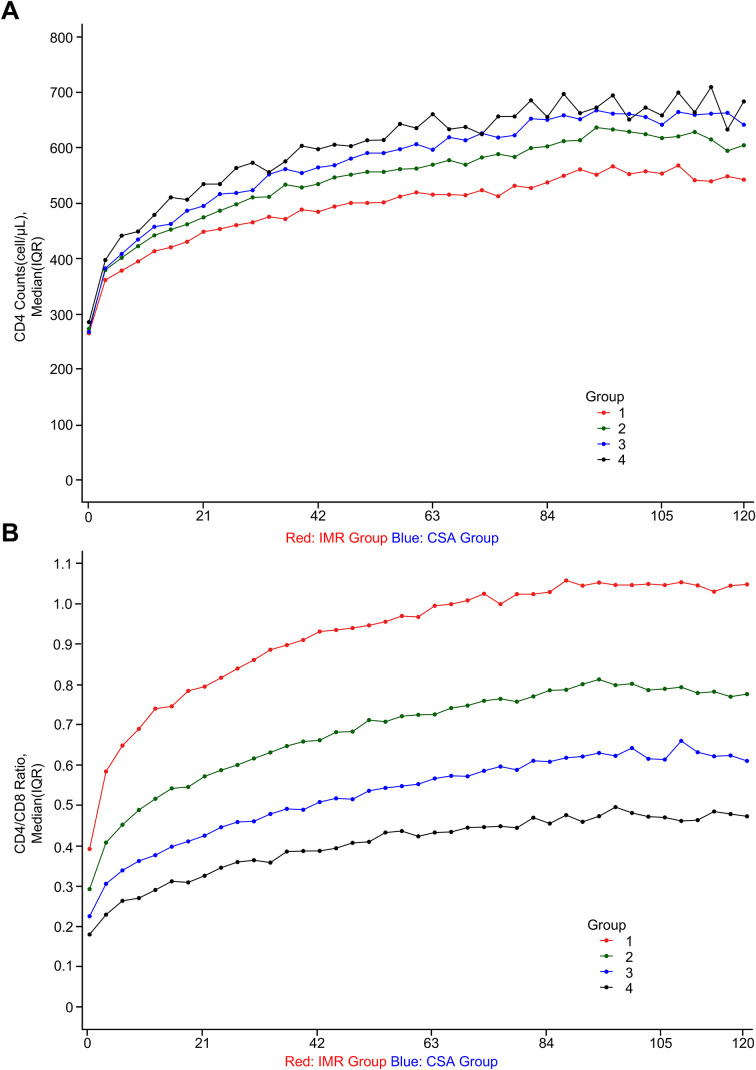
The dynamic trajectory of immune recovery in PLWH. **(A)** The dynamic variation of CD4^+^ T cell counts in PLWH after ART based on the dynamic grouping of CD8^+^ T cell counts. The red curve indicates IMR (n=1,862) and the blue curve indicates CSA (n=1,120). **(B)** The dynamic variation of CD4/CD8 ratio in PLWH after ART based on the dynamic grouping of CD8^+^ T cell counts. The red curve indicates IMR (n=1,862) and the blue curve indicates CSA (n=1,120).

Individuals in the IMR group exhibited decreasing CD8^+^ T cell counts over time, approaching normal ranges by the final follow-up (baseline: 649.5 [IQR, 499–826.5]; last follow-up: 537 [IQR, 436–641]), while those in the CSA group maintained persistently elevated CD8^+^ T cell levels (baseline: 1141 [IQR, 912.75–1420]; last follow-up: 1044.5 [IQR, 868–1229.25]) despite long-term ART (**Figure 1B**, **Table 1**). Both groups had comparable baseline CD4^+^ T cell counts (IMR: 263.5 [IQR, 190–342]; CSA: 266 [IQR, 179.75–365]) and achieved similar levels of CD4^+^ T cell recovery following prolonged ART (IMR: 562 [IQR, 464–698]; CSA: 654.5 [IQR, 515–824]) (**Figure 2A**, **Table 1**). However, their CD4/CD8 ratio trajectories diverged significantly. Initially, both groups had similarly low CD4/CD8 ratios (IMR: 0.39 [IQR, 0.28–0.53]; CSA: 0.225 [IQR, 0.1559–0.316]). By the final timepoint, the ratio had normalized in the IMR group (1.09 [IQR, 0.87–1.39]) but remained persistently inverted in the CSA group (0.637 [IQR, 0.5–0.817]), largely due to sustained CD8^+^ T cell expansion (**Figure 2B**, **Table 1**).

### 3.2 CD8^+^ T cell counts are positively correlated with inflammatory factors

To examine the relationship between immune status and systemic inflammation, we profiled plasma inflammatory cytokines in 46 randomly selected PLWH (IMR = 22; CSA = 24) from the two groups, whose demographic and clinical characteristics are listed in **Supplementary Table S3**. Both IMR and CSA demonstrated substantial increases in CD4^+^ T cell counts during follow-up (**Supplementary Figures S2A, B**). However, as expected based on trajectory classification, the CSA group maintained elevated CD8^+^ T cell counts, whereas the IMR group exhibited a marked decline from baseline, resulting in significantly lower current CD8^+^ T cell levels. Correspondingly, CD4/CD8 ratios improved in both groups over time, but remained significantly lower in the CSA group, reflecting sustained CD8^+^ T cell dominance (**Supplementary Figures S2A, B**).

**Figure 3:**
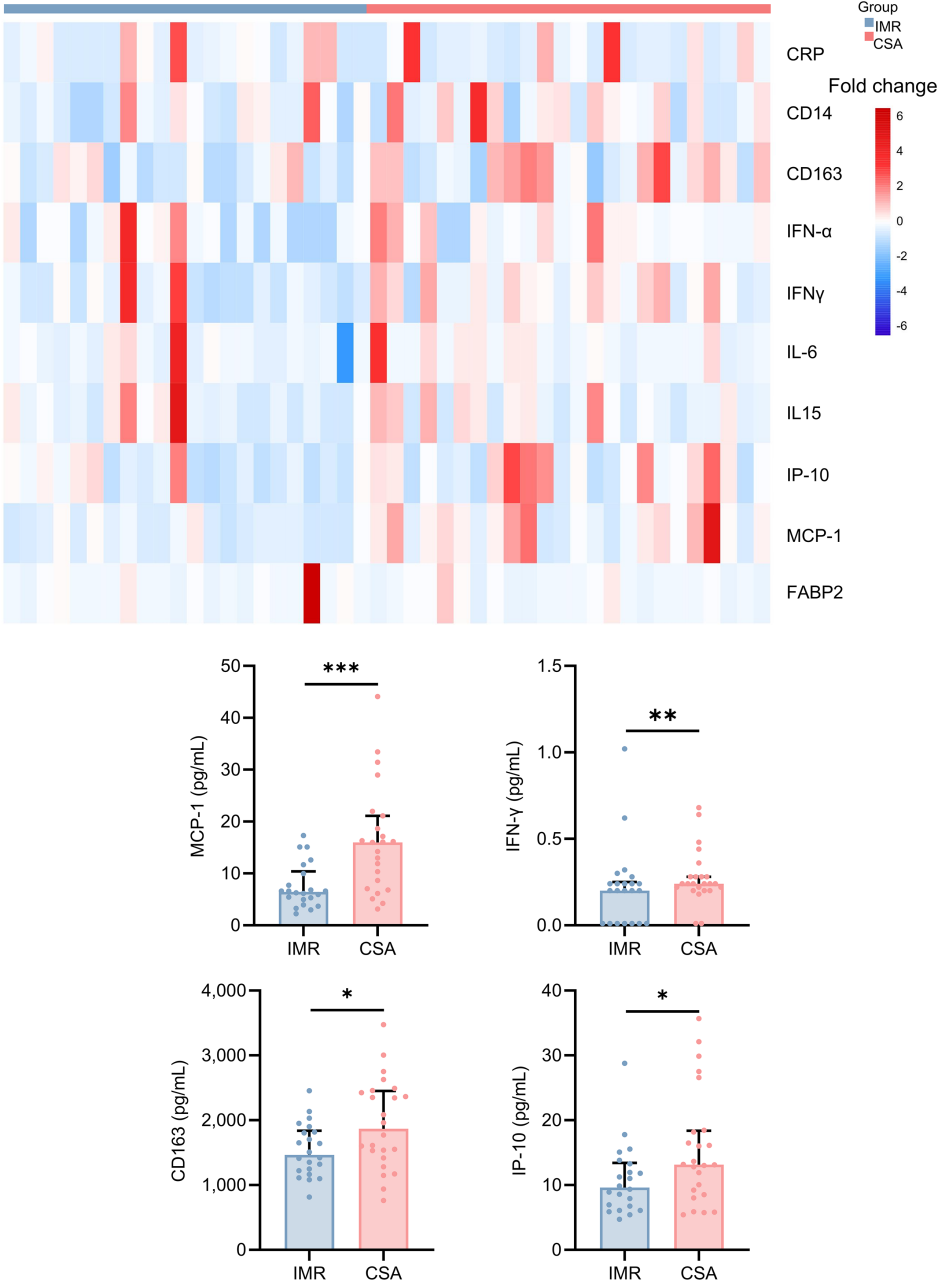
Cytokines profile. IMR (n = 22) and CSA (n = 24) in plasma cytokines represented by the color legend as a change. The heat map shows the expression of cytokines by each participant. For the heatmap, cytokine values were log (x + 1) transformed and z-score normalized. The bar chart shows the cytokines that have significant differences in expression. Mann-Whitney tests were performed to compare the distribution of the variables between groups IMR vs. CSA. Each dot represents one donor. Bars indicate median values; when statistically significant, p-values are indicated as *p < 0.05, ** *p* < 0.01, or ***p < 0.001. CRP, C-reactive protein; IFN, interferon; IL, interleukin; IP-10, interferon-g-inducible protein 10; MCP1, monocyte chemoattractant protein-1; FABP2, fatty acid-binding protein 2.

Cytokines determination revealed that the IMR and CSA groups showed distinct profiles of inflammatory markers and monocyte/macrophage markers. CSA group had significantly higher levels of several key cytokines, including monocyte chemoattractant protein-1 (MCP-1) (p<0.001), IFN-γ (p=0.002), elevated interferon-γ-induced protein 10 (IP-10) (p=0.05), and higher CD163 (p=0.041) (**Figure 3**, **Supplementary Table S4**). No significant differences were observed in CRP, CD14, interferon-α (IFN-α), IFN-β, interleukin-15 (IL-15), CD14, or fatty acid-binding protein 2 (FABP2) (all p>0.05) (**Supplementary Figure S2C**, **Supplementary Table S4**).

### 3.3 CD8^+^ T cells expansion and altered immune composition in the CSA group

**Figure 4:**
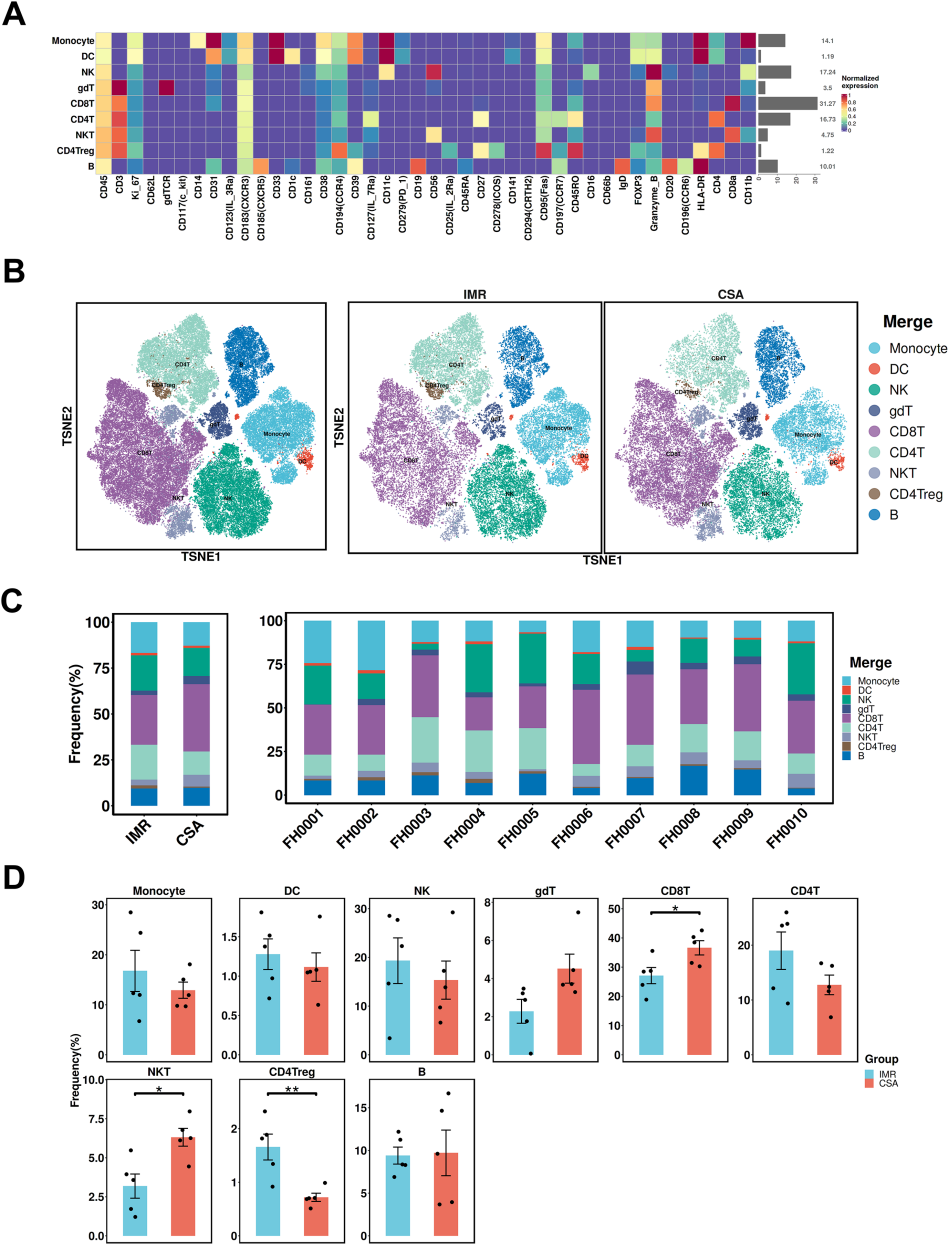
Phenotypical characterization of immune populations in PLWH. **(A)** Heatmap showing the median metal intensity of individual markers for major PBMC immune subsets as indicated. **(B)** t-SNE projection of PBMCs showing major cell populations based on expression of cell type-specific makers. Each dot corresponds to a single cell and colored according to PhenoGraph clustering (left); groups are each colored as indicated (right). **(C)** Comparison of the frequencies of major PBMC immune subsets between the IMR (n = 5) and CSA (n = 5) groups. **(D)** Frequencies of immune cell populations from individuals with IMR (n = 5) and CSA (n = 5). Groups are shown in different colors. Significant differences are indicated by *P < 0.05. Differences between each group were analyzed using a two-sided unpaired Mann–Whitney U-test.

To investigate immune compositional differences after long-term ART, we performed CyTOF on CD45^+^ PBMCs from 10 individuals (IMR, n=5; CSA, n=5), selected from the cytokine-testing cohort based on the availability of sufficient, high-quality cryopreserved PBMCs (**Supplementary Figure S3**). Their key demographic parameters are presented in **Supplementary Table S5**. A 42-marker panel was used to characterize immune differentiation, activation states, and key functional molecules (**Supplementary Table S2**, **Supplementary Figure S4**). Nine major immune subsets were identified using canonical markers: monocytes (CD3^-^CD11b^+^CD14^+^HLA-DR^+^), dendritic cells (CD3^-^CD14^-^CD11b^+^CD11c+HLA-DR^+^), Natural killer (NK) cells (CD3⁻CD56^+^), γδ T cells (CD3^+^γδT^+^), CD8^+^ T cells (CD3^+^CD8^+^), CD4^+^ T cells (CD3^+^CD4^+^), NKT cells (CD3^+^CD56^+^), CD4^+^ regulatory T cells (CD3^+^CD4^+^CD25^+^CD127⁻FoxP3^+^), and B cells (CD3⁻CD19^+^) (**Figures 4A, B**, **Supplementary Table S6**). Compared to the IMR group, CSA participants exhibited significantly higher frequencies of CD8^+^ T cells, γδ T cells, and NKT cells, alongside a marked reduction in CD4^+^ Tregs (**Figures 4C, D**, **Supplementary Table S7**). Compared with the IMR group, the CSA group displayed elevated proportions of cytotoxic and activated immune subsets and reduced proportions of regulatory subsets, as shown in the CyTOF analysis.

### 3.4 In-depth characterization of CD8^+^ T cell clusters

**Figure 5:**
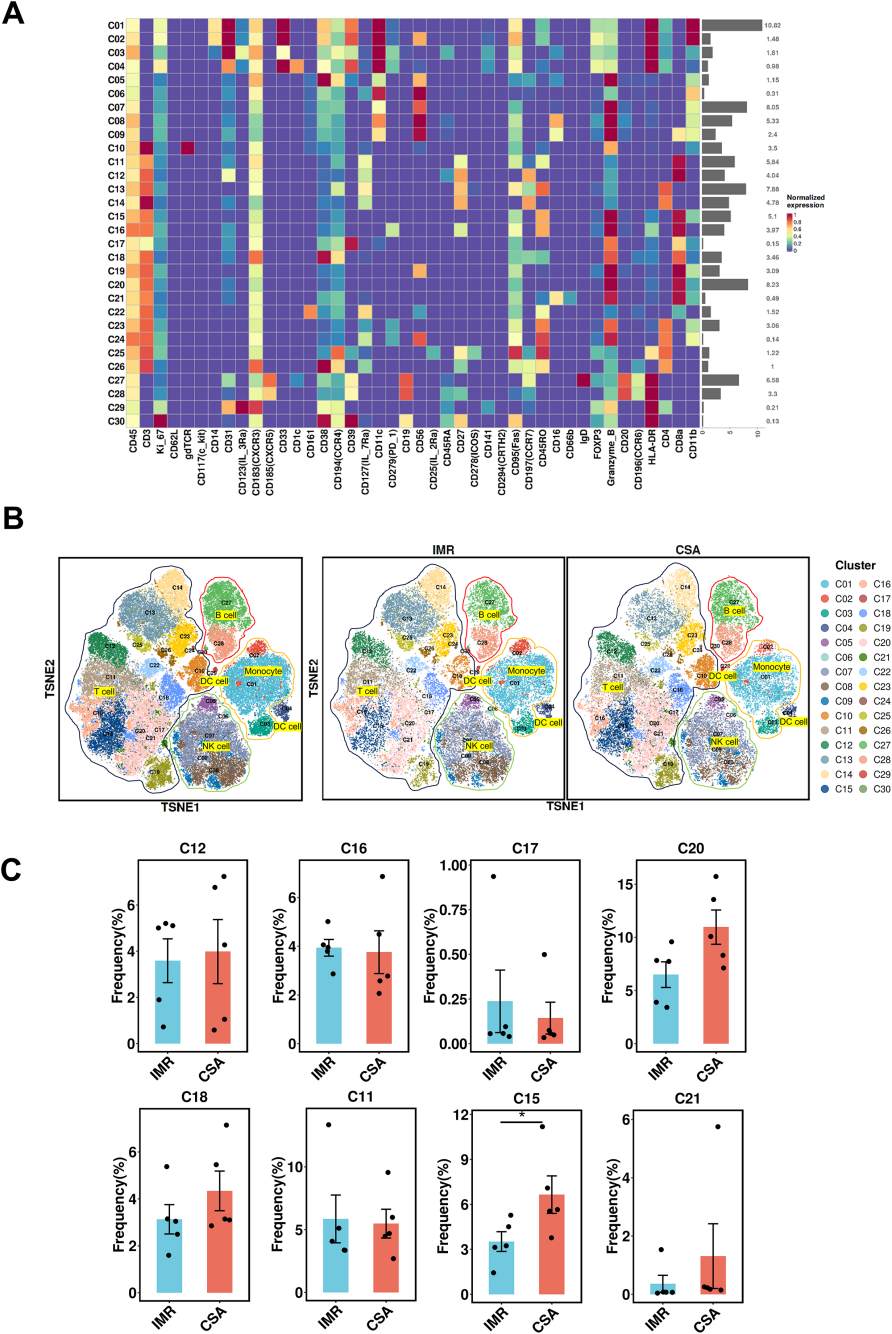
Characterization of immune cell clusters from PBMCs of IMR and CSA. **(A)** Heatmap showing the median metal intensity of individual markers for each cluster as indicated. **(B)** t-SNE projection of PBMCs showing major cell clusters based on expression of cell type-specific makers. Each dot corresponds to a single cell and colored according to PhenoGraph clustering (left); groups are each colored as indicated (right). **(C)** Frequencies of CD8^+^ T cell clusters from individuals with chronic IMR (n = 5) and CSA (n = 5). Groups are shown in different colors. Significant differences are indicated by *P < 0.05. Differences between each group were analyzed using a two-sided unpaired Mann-Whitney U-test.

To further dissect CD8^+^ T cell heterogeneity between IMR and CSA groups, we performed unsupervised clustering on CyTOF data, followed by t-SNE for dimensionality reduction. Thirty immune cell clusters were identified, five of which mapped to canonical CD8^+^ T cell subsets based on surface marker expression (**Figures 5A, B**, **Supplementary Table S8**): (i) Naïve CD8^+^ T cells (C12: CD3^+^CD8^+^CD45RA^+^CCR7^+^), (ii) Exhausted CD8^+^ T cells (C16: CD3^+^CD8^+^PD1^+^), (iii) Effector CD8^+^ T cells (C17, C20: CD3^+^CD8^+^CD45RA^+^CCR7^-^), (iv) Central memory CD8^+^ T cells (C18: CD3^+^CD8^+^CD45RO^+^CCR7^+^) relate to C18, and (v) Effector memory CD8^+^ T cells (C11, C15, C20: CD3^+^CD8^+^CD45RO^+^CCR7^-^) relate to C11, C15 and C21 (**Figures 5A, B**, **Supplementary Figure S5**, **Supplementary Table S9**).

Among these, cluster C15—an effector CD8^+^ T cell subset—was significantly enriched in the CSA group, while other subset frequencies showed minimal intergroup differences (**Figure 5C**, **Supplementary Table S9**). The result showed that C15 as a contributor to CD8^+^ T cell persistence in CSA. However, the lack of major shifts in the other CD8^+^ T cell clusters indicates that the enlarged CD8^+^ T cell compartment in CSA is largely driven by the expansion of this effector subset rather than by a uniform increase across all CD8^+^ T cell phenotypes.

### 3.5 Phenotypic remodeling of CD8^+^ T cells under long-term ART

**Figure 6:**
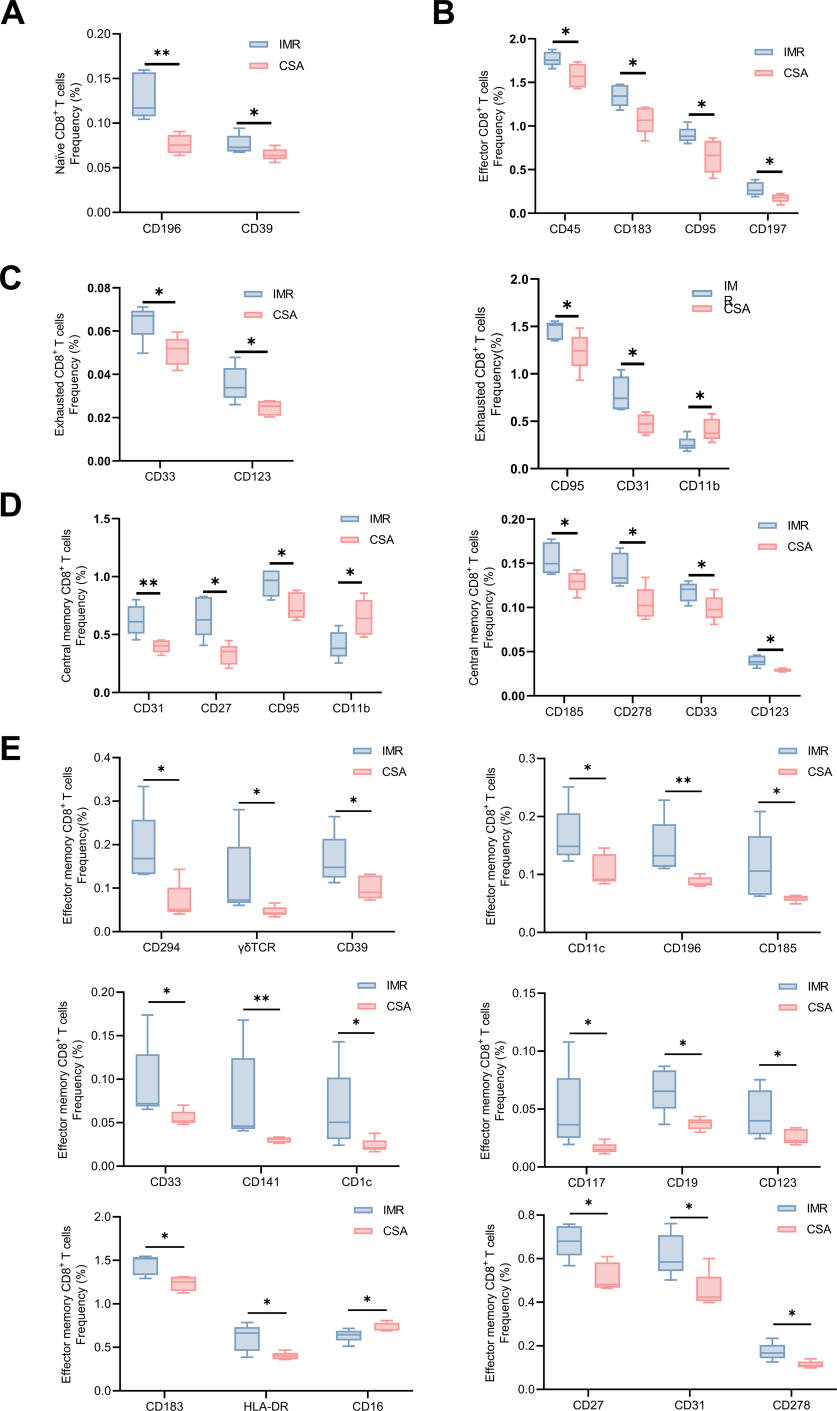
Functional characterization of immune cell subsets in PLWH. Histogram showing the expression distribution of CD8 ^+^ T cell markers of **(A)** naïve CD8^+^ T cell, **(B)** effector CD8^+^ T cells, **(C)** exhausted CD8^+^ T cells, **(D)** central memory CD8^+^ T cells, and **(E)** effector memory CD8^+^ T cells between IMR (n = 5) and CSA (n = 5). Significant differences are indicated by *P < 0.05, or **P < 0.01. Differences between each group were analyzed using a two-sided unpaired Mann–Whitney U-test.

To further investigate phenotypic and functional differences in CD8^+^ T cells between groups, we profiled the expression of 42 immunological markers across CD8^+^ T cell subsets (**Supplementary Table S10**). In the naïve CD8^+^ T cell population, cells from the IMR group exhibited higher expression of CD39 and CD196, markers associated with homeostasis and regulatory potential (**Figure 6A**). Among effector CD8^+^ T cells, IMR samples exhibited elevated levels of CD45, CD183, CD95, and CD197, indicative of preserved activation and migratory competence (**Figure 6B**). Exhausted CD8^+^ T cells from the IMR group showed increased expression of CD31, CD123, CD33, and CD95 (**Figure 6C**), suggesting better maintenance of apoptotic regulation and self-renewal potential. Likewise, central memory CD8^+^ T cells in the IMR group displayed increased expression of CD27, CD185 (CXCR5), CD278 (ICOS), and CD95, supporting a phenotype of sustained costimulatory capacity and follicular homing (**Figure 6D**). Effector memory CD8^+^ T cells in IMR exhibited upregulation of 17 functional markers, further underscoring a more diverse and regulated immunophenotype (**Figure 6E**). Collectively, compared with the IMR group, the CSA group showed higher frequencies of effector-like and exhausted CD8^+^ T cell phenotypes, lower expression of costimulatory and homing molecules, and reduced representation of naïve and central memory–like CD8^+^ T cell subsets.

### 3.6 Transcriptomic profiling reveals enrichment of immune-inflammatory and chemotactic pathways in CSA

**Figure 7:**
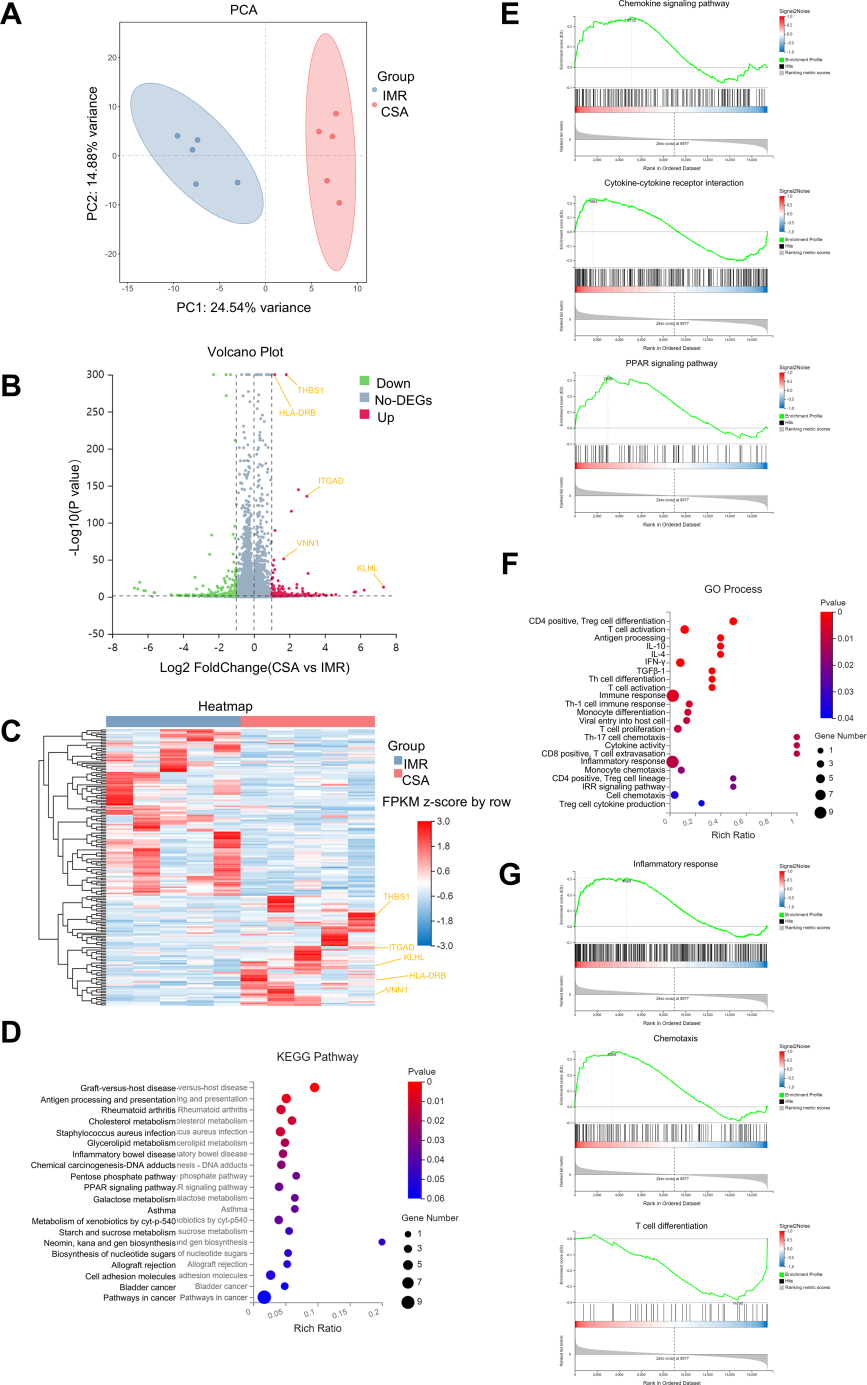
Bulk RNA sequencing indicated that immuno-inflammatory and chemokine pathways were highly expressed in CSA. **(A)** PCA showing the significant difference in gene expression of bulk RNA sequencing between the two groups, with the left side representing 5 cases of PLWH in the IMR group and the right side representing 5 cases of PLWH in the CSA group. **(B)** Volcano plot showing differentially expressed genes between the two groups, with green representing down-regulated genes, red representing up-regulated genes and grey representing no differentially expressed genes. **(C)** Heat map showing the overall level of differentially expressed genes between the two groups. **(D)** KEGG enrichment analysis showing the highest expressed relevant pathways in the CSA group. **(E)** GSEA analysis showing chemokine signaling pathway (left), cytokine-cytokine receptor interaction (middle), and PPAR (right) signaling pathway enrichment results. **(F)** GO enrichment analysis showing the most highly expressed biological processes in the CSA group. **(G)** GSEA analysis showing inflammatory response (left), T-cell differentiation (middle), and chemotaxis (right) enrichment results.

Bulk RNA-seq was performed on PBMCs from the same 10 individuals used for CyTOF profiling (IMR, n = 5; CSA, n = 5). Detailed demographic characteristics of these participants are provided in **Supplementary Table S5**. To elucidate the molecular mechanisms underlying CD8^+^ T cell–driven immune dysregulation in CSA, we performed bulk RNA sequencing on representative IMR and CSA samples (**Supplementary Table S11**). A total of 181 differentially expressed genes (DEGs) were identified, including 72 upregulated and 109 downregulated genes in CSA relative to IMR (**Figures 7A–C**). Significant DEGs were defined using an FDR < 0.001, ensuring high-confidence transcriptional differences between IMR and CSA groups.

Functional enrichment analysis revealed a transcriptional signature in the CSA group marked by immune activation and inflammation. Kyoto Encyclopedia of Genes and Genomes (KEGG) pathway analysis revealed significant enrichment of immune- and inflammation-related pathways in the CSA group, including PPAR signaling and antigen processing and presentation (**Figure 7D**). Gene Set Enrichment Analysis (GSEA) yielded concordant results, highlighting upregulation of immune signaling and cellular interaction pathways (**Figure 7E**). Disease enrichment analysis showed that conditions such as HIV infection and systemic lupus erythematosus were overrepresented in CSA, with GSEA identified enrichment for primary and common variable immunodeficiencies (**Supplementary Figure S6A**). Notably, chemokine signaling genes such as CCR5, CCR2, CXCR1, and TLR3 were markedly upregulated in CSA (**Supplementary Figure S6B**).

Gene Ontology (GO) biological process analysis further underscored the upregulation of cytokine signaling and immune regulation in CSA, including pathways involving T cells, T helper cells, IFN-γ, and interleukins (**Figure 7F**). Ligand–receptor interaction, including CCR2 chemokine binding and CD4 receptor binding, was also significantly enriched, along with pathways involving T cell receptor and cytokine receptor pathways (**Figure 7G**). Consistent with plasma biomarker profiles, elevated expression of chemokines, such as chemokine and their receptor activity and cytokine binding was observed in the CSA group (**Supplementary Figures S6C, D**). Together, these analyses show that, compared with the IMR group, the CSA group has transcriptomic signatures enriched for interferon-stimulated, chemotactic, and inflammatory pathways.

Together, these results indicate that the CSA group is characterized by an immune milieu enriched in interferon-stimulated, chemotactic, and inflammatory signals, which may perpetuate CD8^+^ T cell dysregulation and impede full immunologic restoration despite successful ART.

## 4 Discussion

A subset of ART-treated PLWH maintain elevated CD8^+^ T cell counts and persistently inverted CD4/CD8 ratios, despite achieving viral suppression and CD4^+^ T cell restoration. These individuals remain at elevated risk of chronic immune dysfunction, yet the underlying immunological alterations remain poorly defined (5, 21). In this study, we sought to elucidate the mechanisms of discordant immune recovery by integrating high-dimensional immunophenotyping and transcriptomic profiling. We found that this immunological phenotype (termed the CSA group) is marked by elevated effector and senescent CD8^+^ T cell subsets, downregulated costimulatory and homeostatic markers, and a systemic inflammatory environment dominated by chemokine signaling. These findings suggest that persistent CD8^+^ T cell-driven immune dysregulation is associated with immunological features that have been linked in previous studies to long-term clinical complications in PLWH.

Implications of high CD8 counts despite ART have been widely studied and even associated with clinical progression (22, 23). However, prior studies largely relied on cross-sectional snapshots, limiting insight into how CD8^+^ T cell dynamics shape long-term immune reconstitution. The application of GBTM in this context offers a methodological innovation by enabling a data-driven, clinically interpretable categorization of long-term immune reconstitution upon ART (24, 25). Using GBTM, we captured the temporal dynamics of CD8^+^ T cell recovery in over 5,000 ART-experienced individuals, identifying two distinct recovery patterns (IMR vs. CSA). Notably, baseline viral load was significantly higher in CSA individuals, and over 60% exhibited persistently elevated CD8^+^ T cell counts during follow-up. These data suggest that early antigenic burden may imprint a long-lived proinflammatory immune trajectory, potentially through mechanisms such as residual antigen stimulation, microbial translocation, or bystander activation. Importantly, these findings underscore the predictive value of baseline CD8^+^ T cell counts and viral load to identify patients at risk of immune perturbation beyond CD4^+^ count-based monitoring.

In addition to altered T cell ratios, the CSA group also displayed a distinct systemic inflammatory profile. Plasma levels of MCP-1, IP-10, IFN-γ, and CD163 were significantly elevated. MCP-1 is a potent chemoattractant for monocytes and T cells (26, 27) and has been associated with HIV progression, immune reconstitution failure (28), and cardiovascular disease (29). Elevated IP-10, largely induced by IFN-γ, may further perpetuate inflammation by recruiting activated CXCR3^+^ T cells and innate immune cells (26, 30, 31). CD163 as a marker of monocyte/macrophage activation, has been linked to non-AIDS-defining cancers and mortality (32, 33). These markers collectively reflect ongoing Th1-skewed immune activation and monocyte/macrophage engagement (34, 35). Together, these findings indicate that sustained CD8^+^ T cell expansion is associated with a chronic inflammatory state and altered markers related to vascular and metabolic homeostasis. Although we did not assess clinical endpoints in this study, this pattern is consistent with previous reports linking low CD4/CD8 ratios and monocyte/macrophage activation to higher risks of cardiovascular disease in PLWH.

High-dimensional CyTOF profiling revealed pronounced remodeling of the peripheral immune landscape in CSA patients. Compared to the IMR group, CSA individuals exhibited expansion of CD8^+^ T cells, γδ T cells, and NKT cells, with concomitant reduction in Tregs. This imbalance suggests a shift toward immune activation with impaired immunoregulatory control. NKT cells are known producers of IFN-γ and MCP-1, and their expansion may exacerbate inflammation and CD8^+^ T cell activation (36, 37). Meanwhile, reduced Treg frequency has been implicated in mucosal barrier breakdown and microbial translocation (38), further fueling immune activation. Our findings suggest that the immune landscape of the CSA group is skewed toward cytotoxic activation and diminished regulatory control, which may underpin the observed chronic inflammation and incomplete immune normalization despite CD4^+^ T cell recovery. These compositional changes provide mechanistic insight into how CD8^+^ T cell–driven immune dysregulation is sustained, even in the absence of detectable viremia.

Phenotypic profiling of CD8^+^ T cell subsets revealed widespread downregulation of functional and regulatory markers in the CSA group. The selective enrichment of effector memory CD8^+^ T cells in CSA individuals, particularly cluster C15, may reflect a shift toward more differentiated CD8^+^ T cell phenotypes, a pattern that has been associated with chronic antigen stimulation and immune senescence (39, 40). Moreover, across naïve, central memory, effector, and exhausted CD8^+^ T cell compartments, CSA samples consistently demonstrated downregulation of key functional markers including CD27, CD95, CD183, CD196, and CD278. These markers are critical for maintaining T cell function. CD196 and CD39 promote survival and regulatory functions of naive CD8^+^ T cells (41, 42). CD27 and CD91 maintain costimulatory capacity in CD8^+^ Tcm cells and facilitate thymic input of nascent T cells (43, 44). CD31 and CD95 sustain apoptotic regulation and self-renewal potential in CD8^+^ Tex cells (45, 46). The concurrent downregulation of these markers in CSA indicates that persistent CD8^+^ T cell elevation is accompanied by diminished functional integrity and plasticity (47). Reduced expression of regulatory molecules further suggests diminished immunomodulatory capacity, reinforcing the notion that excessive CD8^+^ T cell proliferation is not functionally beneficial and may perpetuate immune dysregulation (48, 49). This imbalance likely reflects impaired immunoregulation, permitting sustained CD8^+^ T cell activation and chronic inflammation. These features may underlie the increased risk of non-AIDS events and highlight the need for CD8-targeted immune monitoring in long-term ART.

Transcriptomic analysis further revealed dysregulated cytokine and chemokine signaling, immune checkpoint activation, and metabolic adaptation in the CSA group. In particular, we observed upregulation of peroxisome proliferator-activated receptor alpha (PPARα) signaling and lipid oxidation pathways. The upregulation of PPARα and its target genes suggests that activated CD8^+^ T cell lineages rely increasingly on lipid substrates for bioenergetic support (50). This is consistent with previous studies demonstrating that cytotoxic CD8^+^ T cells in chronic viral infections adopt oxidative metabolism to sustain effector function and potentiate the risk of lipid metabolic abnormalities (51, 52). Our results indicate that the CSA group is characterized by an immune milieu enriched in interferon-stimulated, chemotactic, and inflammatory signals, which may perpetuate CD8^+^ T cell dysregulation and impede full immunologic restoration (53, 54). These findings reinforce the biological distinctiveness of the CSA phenotype and suggest that metabolic–inflammatory circuits, such as PPARα pathways or chemokine receptor signaling, warrant further mechanistic investigation in ART-suppressed individuals. Whether modulating these pathways can restore immune homeostasis or reduce non-AIDS comorbidity risk remains to be determined.

Nevertheless, this study has limitations. The mechanistic link between observed immune alterations and specific clinical outcomes requires further validation in prospective cohorts. Additionally, functional assays (e.g., cytotoxicity, cytokine release) were not performed to confirm the effector impairment inferred from transcriptional profiles. What’s more, we did not measure cell-associated HIV DNA or other direct markers of HIV reservoir size in PBMCs. Consequently, we cannot determine whether the CSA phenotype reflects a larger or more active latent reservoir, or instead predominantly host-related immune dysregulation in the context of a similar reservoir burden. Although all participants were receiving long-term ART with virological suppression based on plasma HIV RNA measurements, future studies incorporating standardized assays for cell-associated HIV DNA and replication-competent virus will be required to delineate how reservoir size contributes to divergent immune recovery trajectories.

In conclusion, we define a CD8^+^ T cell–driven immunological phenotype in ART-treated PLWH with discordant immune recovery. Despite CD4^+^ T cell restoration, these individuals exhibit chronic immune activation, regulatory failure, and systemic inflammation. Our findings advocate for a shift from CD4-centric monitoring to integrated immune profiling that includes CD8^+^ T cell dynamics, inflammatory markers, and functional status. This approach may help identify high-risk patients and guide targeted interventions to improve long-term outcomes in the ART era.

## Data Availability

Bulk RNA sequencing data reported in the present paper have been deposited in the NCBI repository (BioProject ID: PRJNA1376969).
