# Supplementary material for: Persistent CD8+ T cell-driven immune dysregulation despite normalized CD4+ T cell recovery in ART-treated people living with HIV
Source: Front Immunol. 2026 Feb 9;17:1735779. doi: 10.3389/fimmu.2026.1735779 (PMC12926161; doi:10.3389/fimmu.2026.1735779)
Supplement: Supplementary file 1 [file DataSheet1.pdf]

A

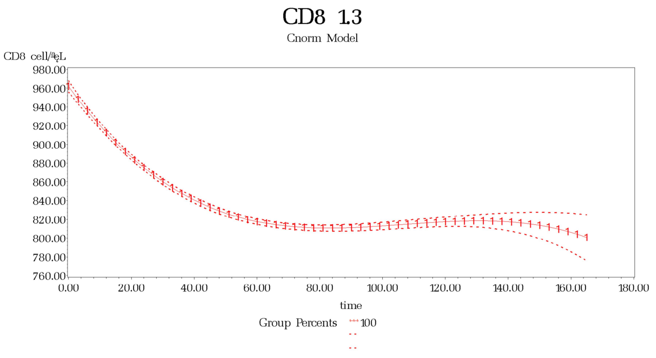

C

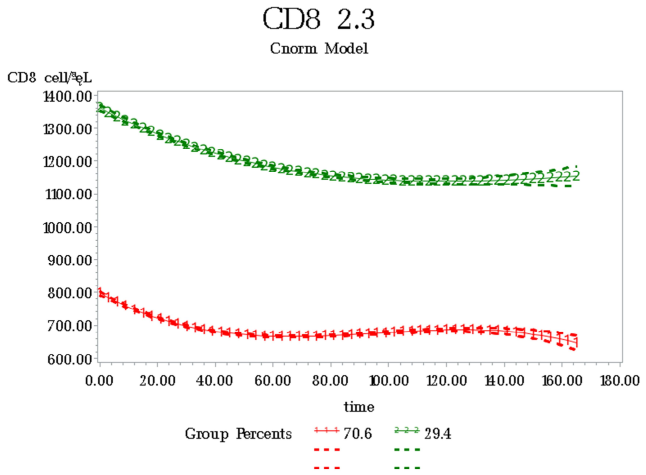

C

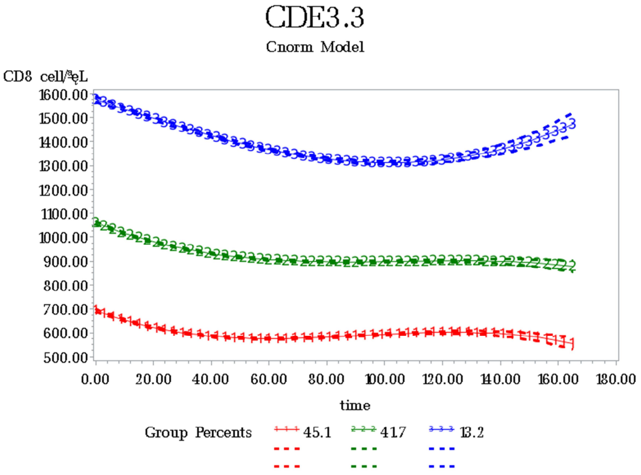

D

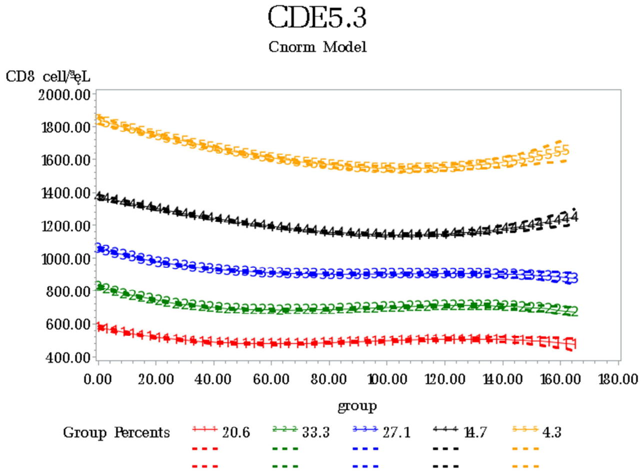

Supplementary Figure S1. The dotted line represents the estimated value of this group, and the solid line represents the actual value of this subgroup. The percentages below each graph represent the proportion of the number of specimens in each subgroup among all the specimens. (A) Trajectory model curve of one group of HIV-infected individuals. (B) Trajectory model curves of the two groups of HIV-infected individuals. (C) Trajectory model curves of the three groups of HIV-infected individuals. (D) Trajectory model curves of the five groups of HIV-infected individuals.

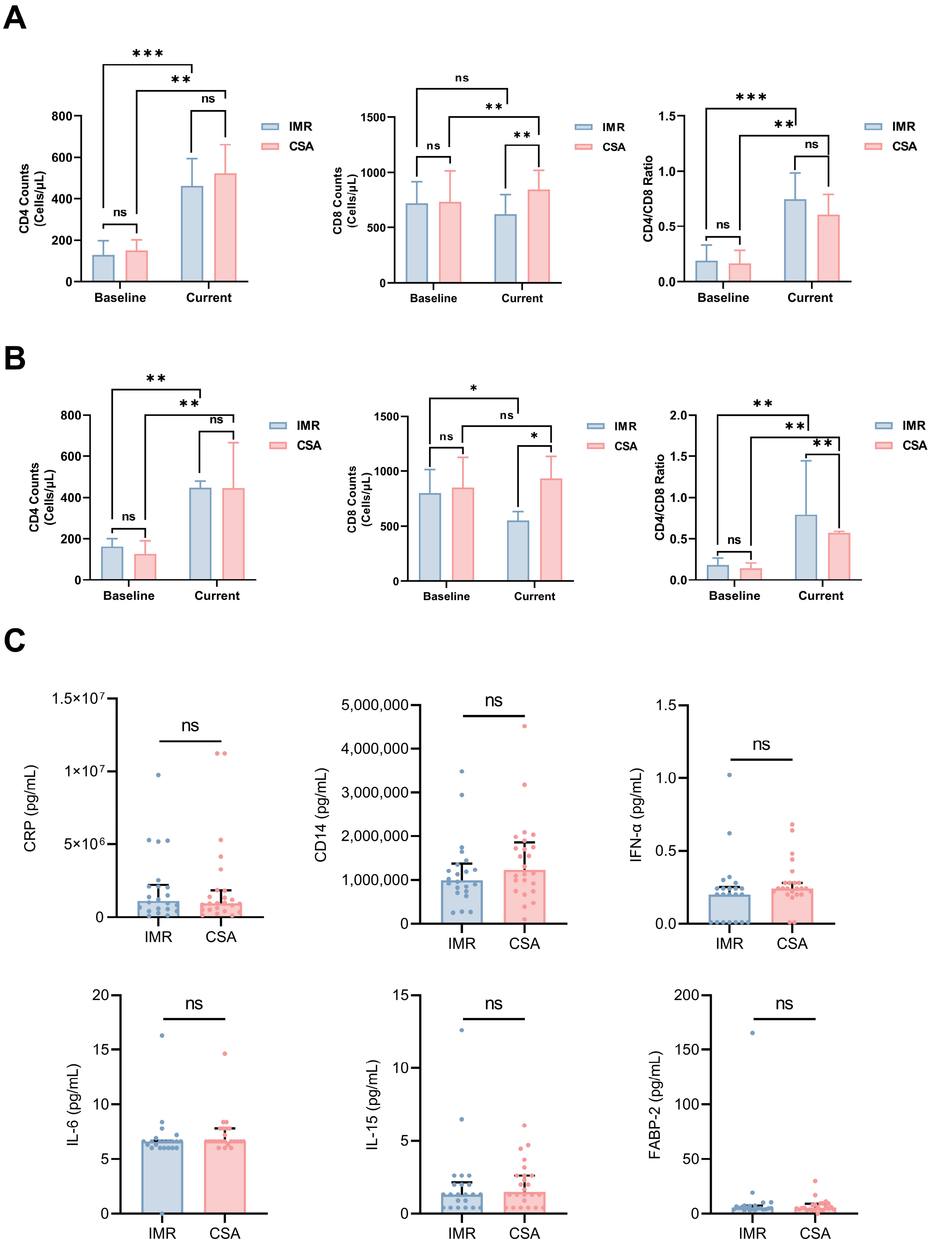

Supplementary Figure S2. (A) FCM detects differences in baseline and last CD4 + T cell counts within and between the two groups (IMR=22, CSA=24) (left). FCM detects differences in baseline and last CD8 + T cell counts within and between the two groups (IMR=22, CSA=24) (middle). FCM detects differences in baseline and last CD4/CD8 ratio within and between the two groups (IMR=22, CSA=24) (right). (B) FCM detects differences in baseline and last CD4 + T cell counts within and between the two groups (IMR=5, CSA=5) (left). FCM detects differences in baseline and last CD8 + T cell counts within and between the two groups (IMR=5, CSA=5) (middle). FCM detects differences in baseline and last CD4/CD8 ratio within and between the two groups (IMR=5, CSA=5) (right). (C) The bar chart shows the cytokines that have significant differences in expression. Mann-Whitney tests were performed to compare the distribution of the variables between groups IMR vs. CSA. *$p < 0.05$, **$p < 0.01$, and ***$p < 0.001$.

A

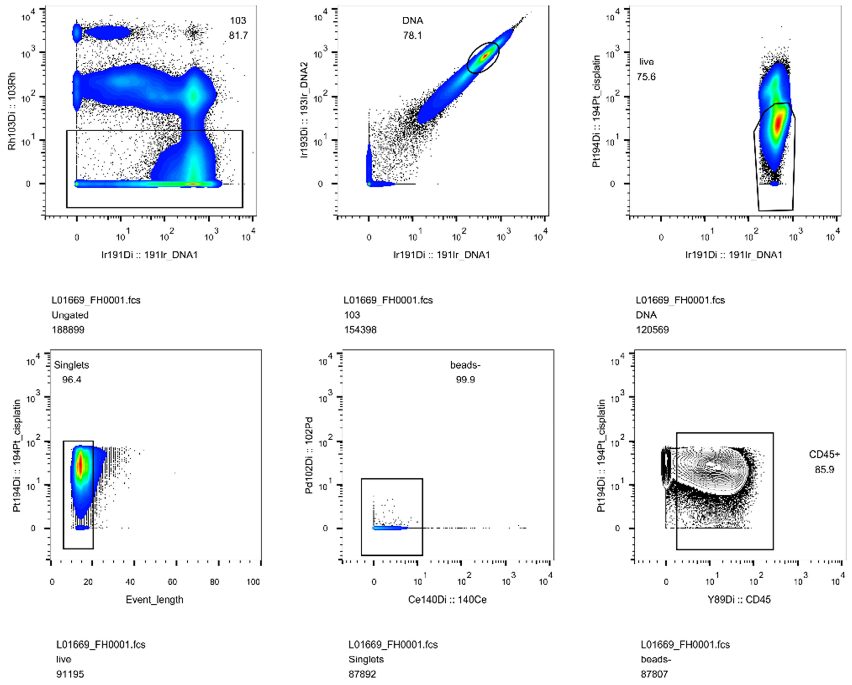

B

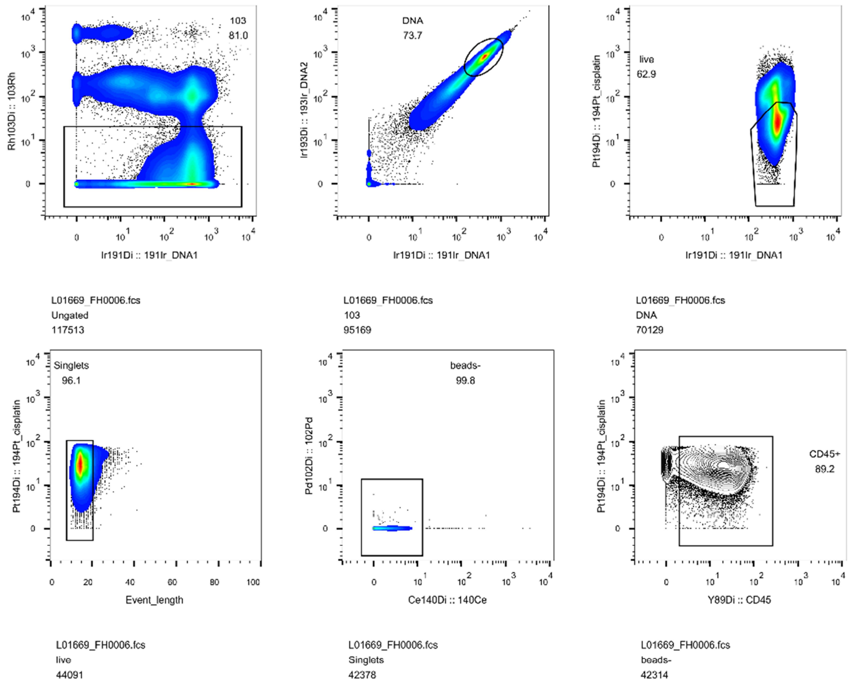

Supplementary Figure S3. Circle gate process of CD45+ cells screened by CyTOF by Cytobank software and FlowJo software (the process from top to bottom, from left to right). (A) based on IMR group PLWH as an example and (B) based on CSA.

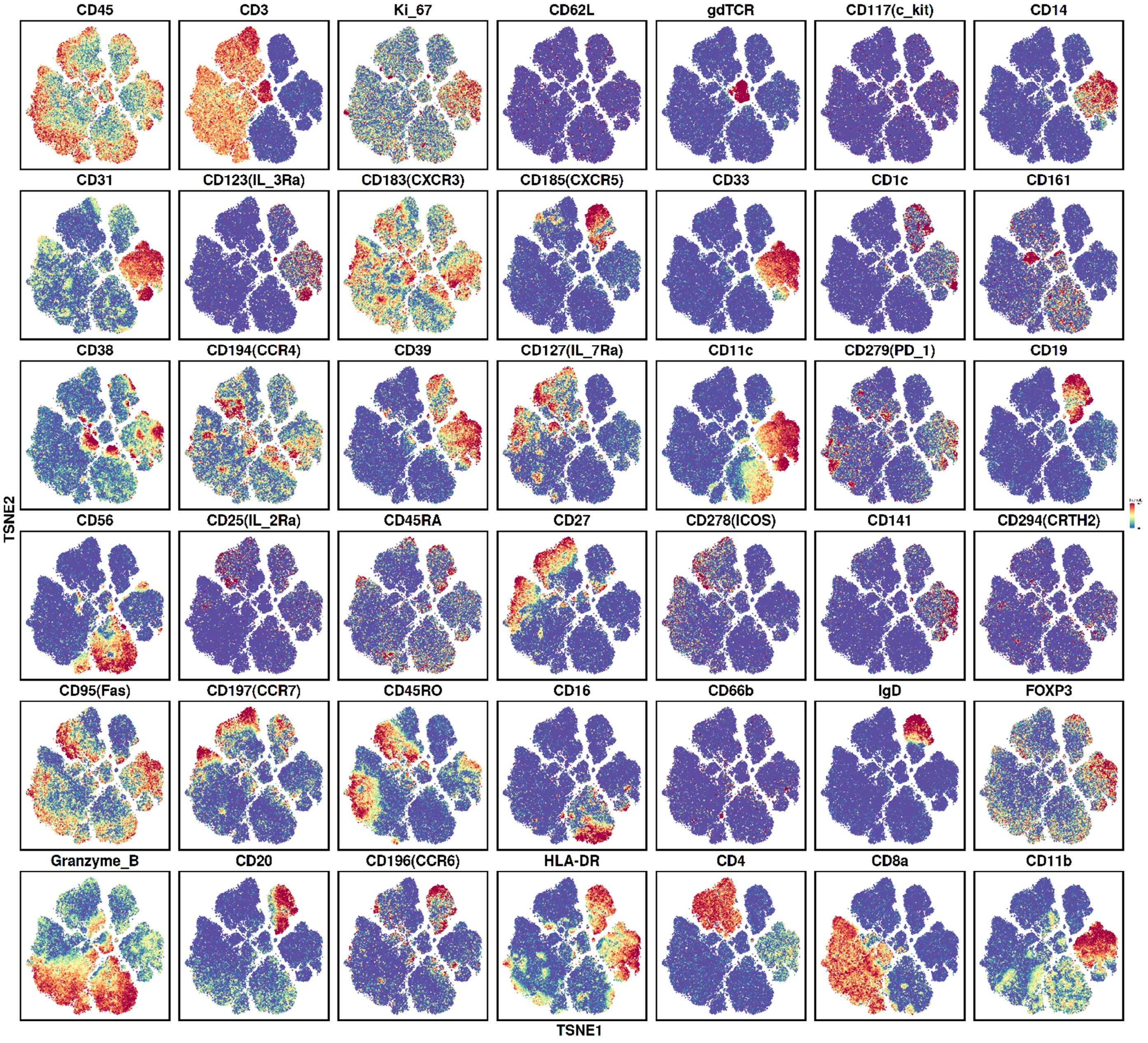

Supplementary Figure S4. T-SNE map showing immune cells from the PhenoGraph cluster identified in 42 biomarkers.

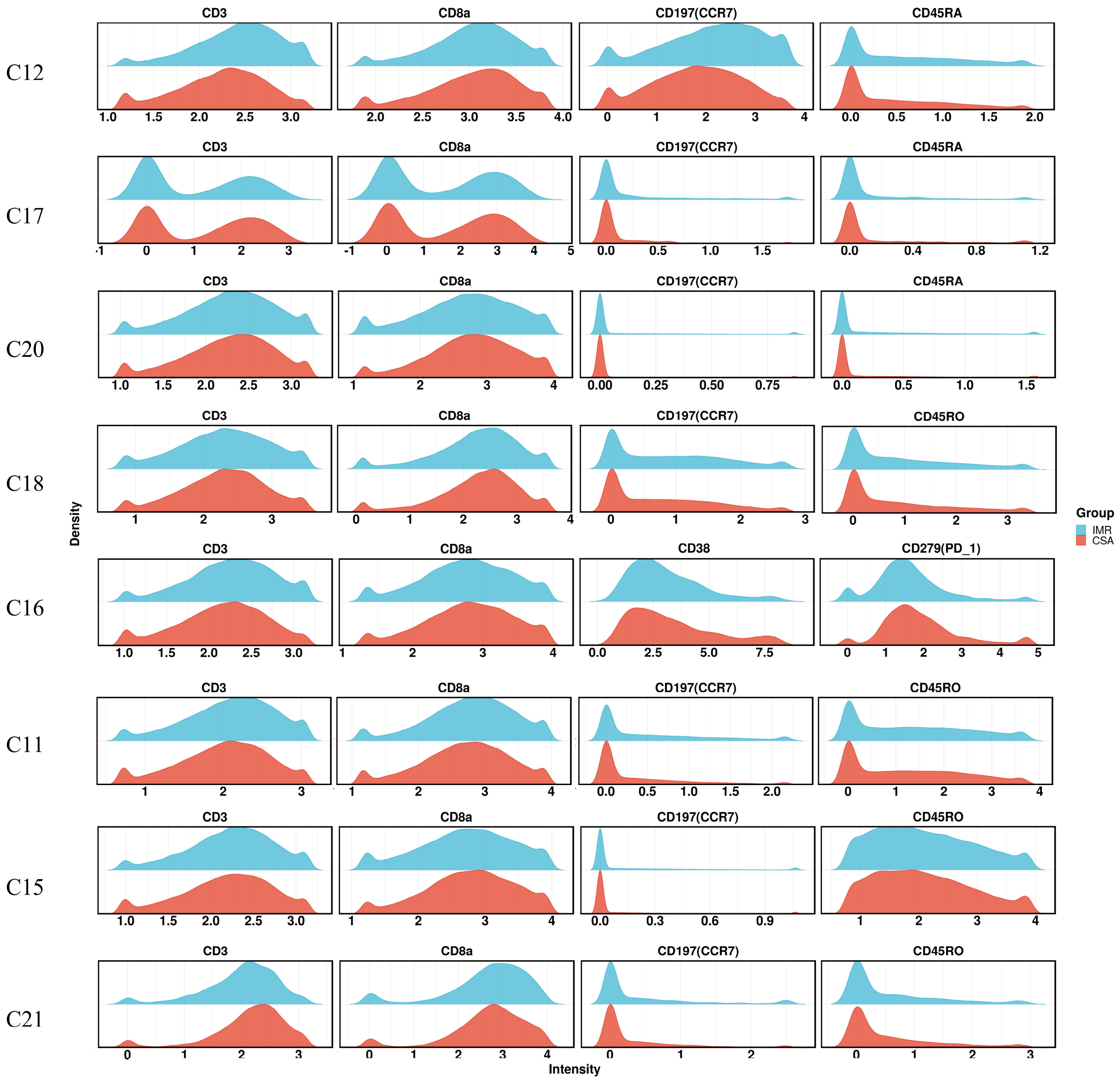

Supplementary Figure S5. Density showing expression of indicated markers for CD8 + T cell clusters, including CD8^{+} Tna (C12), CD8^{+} Tef (C17 and C20), CD8^{+} Tcm (C18), CD8^{+} Tex (C16), CD8^{+} Tem (C11, C15 and C21).

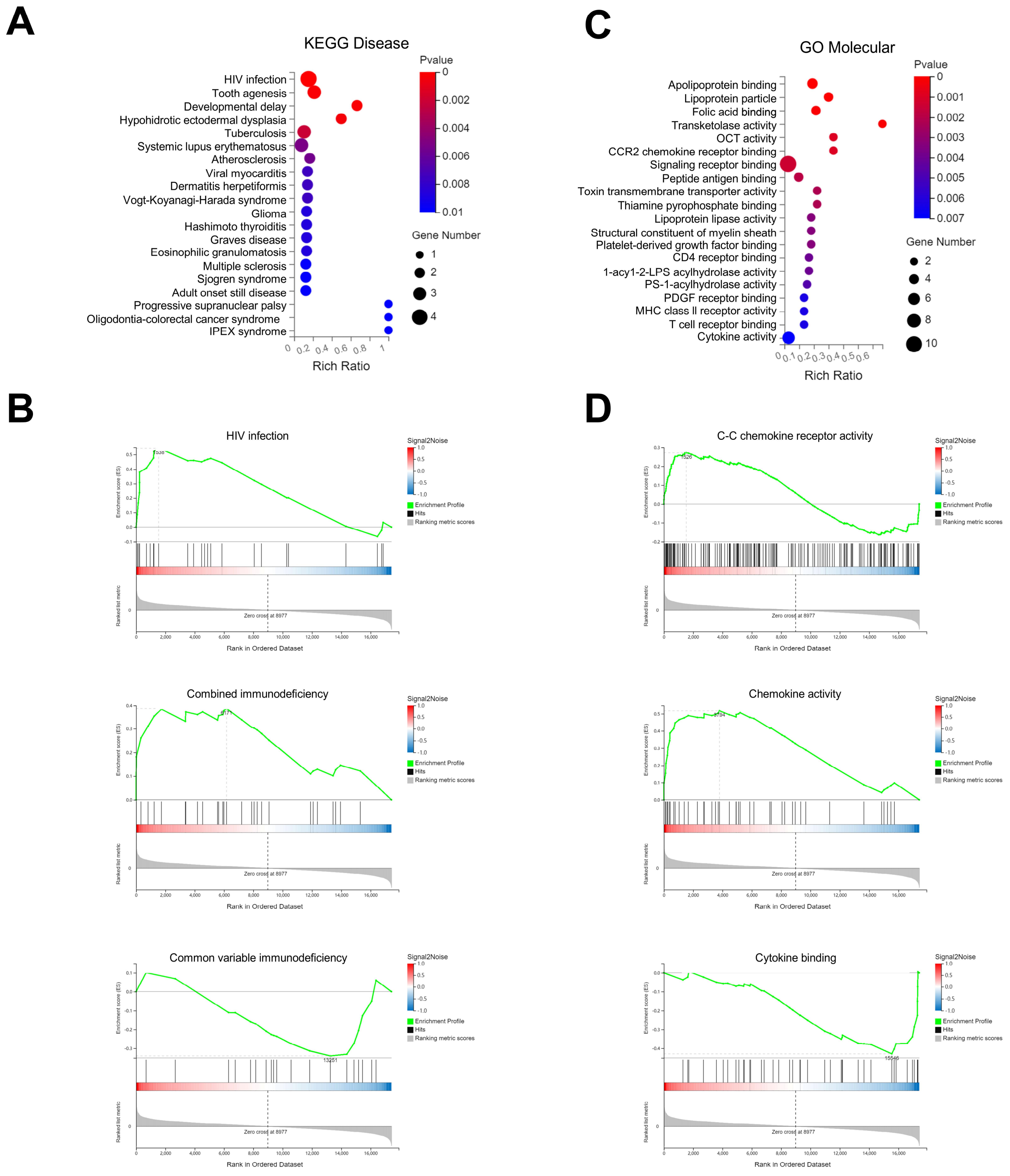

Supplementary Figure S6. (A) KEGG enrichment analysis reveals the highest expressed relevant diseases in the CSA group. (B) GSEA analysis reveals disease enrichment results for combined immunodeficiency (left), HIV infection (middle), and common variant immunodeficiency (right). (C) GO enrichment analysis showing the most highly expressed molecular processes in the CSA group. (D) GSEA analysis showing chemokine receptor activity (left), chemokine activity (middle), and cytokine activity (right) enrichment results.
